# Effects of generating versus evaluating distant analogies on subsequent relational integration: mediating and moderating mechanisms

**DOI:** 10.3389/fpsyg.2026.1902439

**Published:** 2026-08-03

**Authors:** Xuesong Du, Pei Sun

**Affiliations:** 1 Shenzhen Institute of Education Sciences, Shenzhen, China; 2 Department of Psychology, Tsinghua University, Beijing, China; 3 Faculty of Health and Wellness, City University of Macau, Taipa, Macau SAR, China

**Keywords:** cognitive ability, cognitive load, cognitive style, distant analogy generation, relational integration

## Abstract

**Background:**

Relational integration is a core component of higher-order cognition; however, little is known about whether it can be temporarily influenced by prior analogy processing or whether such effects vary across individuals. In the present study, we examined whether generating, rather than evaluating, distant analogies was associated with better subsequent relational integration; whether this association was mediated by cognitive load; and whether it was moderated by individual differences in cognitive abilities and cognitive styles.

**Methods:**

One hundred twenty Chinese college students were randomly assigned to either a distant analogy–generation condition or a distant analogy–evaluation condition. After the priming task, participants completed a multidimensional relational reasoning task (MRRT), a cognitive load measure, and several measures of cognitive ability and cognitive style.

**Results:**

Participants in the generation condition performed better on the MRRT than those in the evaluation condition. This difference was partially mediated by lower cognitive load during the subsequent relational integration task. Working memory capacity substantially moderated the direct association between priming task type and relational integration, whereas fluid intelligence and cognitive style did not moderate this effect to a significant degree.

**Conclusion:**

Overall, the findings suggest that generating distant analogies may temporarily support subsequent relational integration, especially among individuals with relatively lower working memory.

## 1 Introduction

Relational integration is the cognitive ability to mentally connect multiple variables in a way that serves goal-directed behavior and acts as a foundation for a wide range of higher-order cognitive functions, including planning and problem-solving, deductive and inductive reasoning, and analogical and causal inference (Andrews et al., 2006; Goswami, 2013). Thus, effectively enhancing this cognitive process is of paramount importance. Recent studies have confirmed that generating distant semantic analogies can effectively promote relational processing compared with merely evaluating distant semantic analogies or generating near semantic analogies (e.g., Andrews and Bohadana, 2018; Andrews and Vann, 2019; George et al., 2025; Li et al., 2024; Vendetti et al., 2014), with preliminary findings suggesting that cognitive load plays a mediating role in this facilitation effect (Du and Sun, 2022). Examining the individual factors influencing the facilitation effect (whether the generation of distant analogies yields stronger facilitation for some students and weaker for others) will help to further improve the reasoning performance of learners with different characteristics and provide new perspectives for promoting relational integration. In the present study, we addressed these issues by comparing the effects of generating versus evaluating distant analogies on subsequent relational integration while also examining the mediating role of cognitive load and the moderating roles of individual differences.

### 1.1 The facilitation effect of generating distant analogies on relational integration

Relational integration is the process by which multiple explicit representations of relations between entities are jointly considered to draw inferences (Holyoak and Monti, 2021). In the context of analogical reasoning in the A: B: C: D format, relational integration is the operation of comparing the first-order relation between A and B with that between C and D, holding both relations in working memory, and evaluating the higher-order similarity that binds the two pairs. Discussions on how to facilitate relational reasoning have traditionally centered on ability-based facilitation, which aims to permanently enhance an individual’s capacity to process relations through long-term training or accumulated experience (Simms et al., 2018). In contrast, state-based facilitation, which refers to the transient tendency to notice and prioritize relational information when solving a given task, has received far less empirical attention. Bliznashki and Kokinov (2010) first demonstrated that completing a task that implicitly engages participants in relational analysis can lead them to adopt a more relational processing style in a subsequent, unrelated task, suggesting that cognitive states can be temporarily shifted without sustained training. Building on this insight, we argue that short-term, state-based facilitation of relational reasoning offers greater flexibility, lower implementation costs, and higher practicality in educational contexts than ability-based approaches (Du and Sun, 2022; George et al., 2025).

Analogical problems vary along a continuum of semantic distance, which refers to the degree of conceptual overlap or dissimilarity between the source and target domains. In near analogies, the source and target domains share salient surface features or belong to closely related categories, allowing for the direct matching of identical relational structures. For example, the near analogy “furnace: coal: woodstove: wood” can be solved by directly applying the same “fuel-burning” relation from the first pair to the second. In contrast, distant analogies require the reasoner to go beyond surface similarities and identify a higher-order, more abstract relation that spans semantically dissimilar domains. For instance, the distant analogy “furnace: coal: stomach: food” demands the abstraction that both devices “process input materials through internal transformation,” a relation that is not immediately evident from the surface features of the terms. Thus, solving distant analogies imposes greater demands on relational integration than solving near analogies.

Prior work suggests that the facilitation effect depends not simply on semantic distance but rather on the combination of distance and active generation. Generating distant analogies may enhance later relational integration because doing so requires individuals to actively construct, maintain, and compare higher-order relational mappings, thereby increasing the likelihood that subsequent processing will be guided by relational structure rather than featural similarity. Consistent with this view, Vendetti et al. (2014) first demonstrated in a seminal study that participants who generated solutions to distant analogies were subsequently more likely to select relational over featural matches in an unrelated picture-mapping task, whereas participants who evaluated complete analogies showed no such boost. Furthermore, by pooling conditions from their Experiments 1a (evaluation) and 1b (generation), Vendetti et al. (2014) found that the facilitation-transfer effect was uniquely tied to generating distant analogies (rather than evaluating them or generating near analogies). In other words, semantic distance alone was insufficient; the generative component was equally essential. Du and Sun (2022) systematically examined the joint effects of semantic distance (near vs. far vs. control) and priming task type (evaluation vs. generation) using two classical relational integration tasks: the n-term premise integration task (which requires constructing an ordered sequence from relational premises) and the Latin square task (which requires completing a matrix under relational constraints). Across Experiments 1a and 1b, Du and Sun consistently found that neither “distant analogy” alone nor “generation” alone could reliably facilitate relational integration. Instead, the facilitation effect emerged only when both conditions were met simultaneously. Collectively, these findings converge on the conclusion that generating only distant analogies reliably facilitates subsequent relational reasoning.

Most previous studies have conceptualized the facilitation effect as the advantage of generating distant analogies over generating near analogies. The present study examines a complementary contrast: when semantic distance is held constant at the distant level, does generating analogies lead to better relational integration than simply evaluating them? This comparison is theoretically important because it isolates the role of active relational construction from that of semantic distance, thereby allowing a clearer test of whether the constructive act of generating a relation—rather than merely recognizing one—drives the facilitation effect on subsequent relational reasoning.

### 1.2 The mediating role of cognitive load

The generation of novel relational mappings may reduce the mental effort required for subsequent reasoning tasks. According to Cognitive Load Theory (Sweller et al., 2019), working memory resources are limited, and learners’ cognitive processes are subject to three distinct types of load: intrinsic, extraneous, and germane cognitive load. Intrinsic cognitive load refers to the cognitive demand imposed by the inherent complexity of the material to be learned, which is determined by the number of interactive elements that must be processed simultaneously (Sweller, 2010; Sweller et al., 2019). For example, solving a four-term analogy problem—which requires integrating relations across two sets of terms and mapping the relational structure—engages a higher level of element interactivity than simpler tasks and thus imposes a greater intrinsic load. Extraneous cognitive load arises from the manner in which information is presented to learners; suboptimal instructional designs or irrelevant distracting information increase this type of load without contributing to learning (Sweller et al., 2019). In the context of the present study, the stimulus materials and presentation interface were identical across both experimental conditions, thereby maintaining a constant extraneous load. Germane cognitive load represents the cognitive resources that learners actively allocate to schema construction, abstraction, and the deeper integration of knowledge (Klepsch et al., 2017; Sweller et al., 2019). A higher germane load reflects more effective learning processes. Importantly, these three types of cognitive load are additive: when the extraneous load is reduced, more cognitive resources become available for germane processing, which, in turn, facilitates schema construction and knowledge integration.

Investigations on how generating distant analogies influences these cognitive load components have been initiated by researchers. In a comprehensive study, Du and Sun (2022; Experiments 2 and 3) demonstrated that generating distant analogies substantially reduced cognitive load for a subsequent relational integration task compared with evaluating distant analogies, with cognitive load serving as a mediating factor in this facilitation effect. In contrast to passive analogy evaluation, active analogy generation engages individuals’ prior knowledge of relationships. This engagement facilitates the integration of new information with existing knowledge, thereby reducing the cognitive load typically required by working memory and enhancing the efficiency of relational reasoning. Building on this theoretical account, we aimed to re-examine the mediating role of cognitive load in the facilitation effect of generating (vs. evaluating) distant analogies on relational integration. This account does not imply that generating distant analogies is necessarily less effortful than evaluating them during the priming phase itself. Instead, it implies that the constructive processing required during generation may better prepare participants for the later relational integration task, thereby reducing learners’ cognitive load of that subsequent task.

### 1.3 The influencing factors of relational integration

One of the distinctions between students with good and poor academic performance is their ability to efficiently generate relational schemas and engage in relational processing (McDaniel et al., 2014). Understanding why some learners benefit more from relational priming than others carries important implications for theory and educational practice. When the amount of knowledge required to solve relational reasoning problems remains constant, performance on relational reasoning tasks is closely related to individual differences in cognitive resources (Richland et al., 2006; Simms et al., 2018; Thibaut et al., 2010). Relational Complexity Theory posits that cognitive abilities, such as fluid intelligence and working memory, are crucial for the development of relational reasoning (Halford, 2005). Indeed, higher fluid intelligence (Bhandari and Duncan, 2014; Birney and Bowman, 2009) and greater working memory capacity (Copeland and Radvansky, 2004; Oberauer et al., 2005) have both been associated with better relational reasoning performance.

These same cognitive capacities may also shape who benefits most from analogy-based facilitation. Prior studies suggest that priming strategies that support relational processing may be more beneficial for individuals with lower cognitive abilities. For example, Vendetti et al. (2014) found that analogy generation improved relational integration performance primarily among individuals with lower fluid intelligence, with little benefit for those with higher fluid intelligence. Similarly, Kubricht et al. (2017) showed that animation-based support facilitated spontaneous transfer, primarily among participants with lower fluid intelligence. According to Cognitive Load Theory (Sweller et al., 2019), individuals with lower working memory capacity are more susceptible to cognitive overload when processing complex relational information; consequently, any manipulation that reduces cognitive load—such as generating distant analogies—should yield greater benefits for these individuals than for those with ample cognitive resources. Thus, we expected the facilitative effect of generating distant analogies to be most pronounced among individuals with lower cognitive abilities, whereas those with higher abilities would receive little additional benefit.

Beyond cognitive abilities, recent studies have consistently shown that cognitive styles (preferred thinking strategies) also influence relational reasoning performance (e.g., Gray and Holyoak, 2020; Grossnickle et al., 2016). Cognitive reflection refers to the tendency or ability to inhibit an intuitive but incorrect response and engage in further analytic thinking (Frederick, 2005). Because relational integration often requires suppressing salient but superficial associations in favor of higher-order relational structure, cognitive reflection may be relevant to the extent to which individuals benefit from analogy-based facilitation. In the present study, we used a seven-item version of the Cognitive Reflection Test (Šrol, 2018; Toplak et al., 2014), which has shown acceptable psychometric properties in prior research.

We also explored two broader motivational-epistemic styles: need for cognition and need for cognitive closure. There exists a moderate and positive correlation between the need for cognition and relational reasoning (Fleischhauer et al., 2010). The need for cognition reflects the degree to which an individual actively seeks out and derives enjoyment from engaging in activities that necessitate cognitive effort (Cacioppo and Petty, 1982; Cacioppo et al., 1984) and is regarded as a domain-general, intrinsically individual trait. When completing tasks with high cognitive demands, individuals with high levels of need for cognition tend to report more positive experiences and are willing to expend more effort and time (Enge et al., 2008). In contrast, those with lower levels of need for cognition tend to avoid thinking activities and are less likely to process information in detail. However, because direct evidence linking the need for cognition to short-term relational priming remains limited, its role in the present study was treated as exploratory.

The need for cognitive closure reflects a preference for certainty and a desire for definite answers, often accompanied by reduced tolerance for ambiguity (Kruglanski, 2013). Individuals with a high need for cognitive closure exhibit a low tolerance for uncertainty and tend to anticipate finding definitive answers as quickly as possible, even when the information is inadequate. Conversely, those with a low need for cognitive closure are highly motivated to acquire knowledge that aids their reasoning processes, and they typically do not feel rushed to arrive at conclusive answers. Because relational reasoning can require considering alternative interpretations and maintaining uncertainty during integration, this construct may be relevant to performance. However, given the limited prior evidence connecting the need for cognitive closure to short-term analogy-based facilitation, its role in the present study was likewise exploratory.

Based on these insights, the present study aims to systematically examine whether the facilitation effect of generating (vs. evaluating) distant analogies on relational integration is moderated by individual differences in cognitive abilities (fluid intelligence and working memory capacity) and cognitive styles (cognitive reflection, need for cognition, and need for cognitive closure). Specifically, we tested whether these moderators influenced the direct pathway from priming task type to relational integration performance while also re-examining the mediating role of cognitive load in this facilitation effect. The findings are expected to provide empirical evidence for designing priming tasks that are better tailored to learners’ diverse cognitive characteristics.

The present study makes three specific contributions beyond previous research. First, it moves beyond establishing the facilitating effect of distant analogy generation on relational integration to testing the mediating role of cognitive load, thereby providing a more rigorous account of the underlying mechanism. Second, it examines for whom this effect is most pronounced by testing multiple moderators, including fluid intelligence, working memory capacity, cognitive reflection, need for cognition, and need for cognitive closure. Third, by integrating mediation and moderation into a single analytical framework, the study offers a more comprehensive account of the cognitive mechanism and boundary conditions of analogy-based facilitation in relational integration. Based on the theoretical rationale and empirical evidence reviewed above, the present study proposes the following hypotheses:

*Hypothesis 1 (H1)*: Compared with evaluating distant analogies, generating distant analogies will (a) improve participants’ performance on the relational integration task and (b) reduce their cognitive load.

*Hypothesis 2 (H2)*: Cognitive load will mediate the facilitation effect of generating distant analogies on relational integration.

*Hypothesis 3 (H3)*: Fluid intelligence will moderate the direct path of the mediation model. Specifically, generating distant analogies will substantially enhance relational integration for individuals with low fluid intelligence but will not produce a substantial improvement for those with high fluid intelligence.

*Hypothesis 4 (H4)*: Working memory capacity will moderate the direct path of the mediation model. Specifically, generating distant analogies will substantially enhance relational integration for individuals with low working memory capacity but will not produce a substantial improvement for those with high working memory capacity.

*Hypothesis 5 (H5)*: Cognitive reflection will moderate the direct path of the mediation model. Specifically, generating distant analogies will substantially enhance relational integration for individuals with low cognitive reflection but will not produce a substantial improvement for those with high cognitive reflection.

*Hypothesis 6 (H6)*: The need for cognition may moderate the direct path of the mediation model, although this prediction is more exploratory because prior evidence directly linking this construct to short-term relational priming effects is limited.

*Hypothesis 7 (H7)*: The need for cognitive closure may moderate the direct path of the mediation model, although this prediction is likewise exploratory given the limited prior evidence in this specific context.

It is important to distinguish between the present study’s theory-driven hypotheses and its exploratory analyses. For fluid intelligence, working memory capacity, and cognitive reflection, prior theoretical frameworks and empirical findings provide clear grounds for directional predictions (H3–H5). In contrast, the existing evidence is insufficient to support strong directional predictions for need for cognition and need for cognitive closure. We therefore treat these two analyses as exploratory (H6–H7), aiming to generate rather than confirm hypotheses for future research.

## 2 Methods

### 2.1 Participants

We converted the η^2^ value (0.15) provided in previous literature (Vendetti et al., 2014) to Cohen’s *d* (resulting in 0.84) at https://www.psychometrica.de/effect_size.html. In PANGEA^1^, the effect size was set to Cohen’s *d* = 0.84, with two groups. If the number of subjects in each group was 40, the calculated power was 0.96. We recruited 120 college students aged 18–27 years [*M* = 20.76, standard deviation *(SD)* = 2.18], including 65 men and 55 women, and randomly assigned them to the distant analogy generation group (*n* = 59) or the distant analogy evaluation group (*n* = 61). The experiment received approval from the institutional review board of the Department of Psychology at Tsinghua University. Participation was voluntary, and participants could choose to receive either course credit (for psychology undergraduates) or a monetary payment as compensation. Participants provided their written informed consent to participate in this study.

### 2.2 Study design

A one-way between-subjects design was employed, focusing on two types of priming tasks: distant analogy generation and distant analogy evaluation. In this study, the type of priming task served as the independent variable, whereas the score on the relational reasoning task acted as the dependent variable. Meanwhile, the cognitive load score functioned as a mediating variable. Additionally, fluid intelligence, working memory, and cognitive style—encompassing cognitive reflection, the need for cognition, and the need for cognitive closure—were identified as moderators.

### 2.3 Materials

#### 2.3.1 Priming task

##### 2.3.1.1 Distant analogy–generation task

The materials for the distant analogy–generation task mainly came from Green et al. (2010). These investigators used latent semantic analysis (Landauer et al., 1998) to measure the semantic distance between word pairs^2^. To account for potential cultural differences, 30 undergraduates who did not participate in the main experiment were recruited to rate the items from Green et al. (2010). Each item was evaluated on two dimensions using a 7-point Likert scale: word familiarity (1 = *very unfamiliar*, 7 = *very familiar*) and the rationality of the word pair relations (1 = *very unreasonable*, 7 = *very reasonable*). Only items with mean scores above 5 points on both dimensions were retained as experimental materials. This selection procedure excluded 13 items from the original set of 80. Additionally, six graduate students were asked to create some analogy reasoning items as supplements (with familiarity and rationality scores of >5 points and with trying to keep the latent semantic analysis scores consistent with the original items). Combining these two approaches, we finally determined 40 distant analogy generation items for the formal experiment, each presented in the form of “A: B: C: _,” such as “fireplace: coals: stomach: _” (the correct answer is “*food*”). The analogies were presented online via a survey platform (Wenjuanxing, https://www.wjx.cn/), and participants provided written responses. First, two practice items were presented, each followed by subsequent feedback collection, to ensure comprehension of the task requirements, whereas the remaining 40 items served as the test problems. A response (the D term) was considered correct if it matched the solution reported by Green et al. (2010) or if, together with the A, B, and C terms, it formed a valid analogy. The maximum possible score was 40 points.

##### 2.3.1.2 Distant analogy–evaluation task

The materials for the distant analogy–evaluation task were basically the same as those for the distant analogy–generation task, with the difference being that the presentation form was “A: B: C: D.” The items (A: B) in the first half of the distant analogy evaluation task were the same as those in the distant analogy–generation task, but those in the second half (C: D) were different, such as “fireplace: coals: stomach: food.” There were 40 items in the distant analogy–evaluation task (30 valid analogies and 10 invalid analogies). All items were presented via the same online survey platform, and participants were asked to determine whether the relations of “A: B” and “C: D” were consistent. The experiment began with two practice items, each followed with subsequent feedback collection to ensure participants understood the instructions. Then, participants proceeded to the main experiment, which consisted of 40 questions. The total score for the task was 40 points.

#### 2.3.2 Multidimensional relational reasoning task

Cortes et al. (2021) previously designed the multidimensional relational reasoning task (MRRT), which can systematically vary a series of stimulus properties (number of premises, number of dimensions, relation type, and premise order) in a set of relational reasoning problems. Therefore, the MRRT was chosen as the relational integration task for this study. Two graduate students majoring in English and the experimenter translated the questions into Chinese. Each question consisted of two to three premises and one conclusion. For example, two premises are presented on the screen: “*Logan is to the right of Lucas*” and “*Peter is to the right of Logan*.” The participants need to integrate and analyze the relational information in the premises and judge whether the conclusion “*PETER IS TO THE RIGHT OF LUCAS*” is correct (the correct answer is “*True*”). If the conclusion could be necessarily inferred from the premises, participants were required to answer “True,” whereas, if the conclusion was wrong or uncertain, they were required to answer “False.” Taking into account factors such as the duration of the experiment and the difficulty of the task, we selected 60 questions for the final experiment; therefore, the total score for MRRT was 60 points. The MRRT showed acceptable internal consistency (Cronbach’s *α* = 0.84).

#### 2.3.3 Cognitive load

We used the scale developed by Klepsch et al. (2017), noted for its reliability and validity. This scale assessed three dimensions: intrinsic cognitive load (2 items, e.g., “*For this task, many things need to be kept in mind simultaneously*”), extraneous cognitive load (3 items, e.g., “*During this task, it was exhausting to find the important information*”), and germane cognitive load (2 items, e.g., “*I made an effort, not only to understand several details, but to understand the overall context*”), each measured on a 7-point Likert scale (1 = *completely wrong*, 7 = *absolutely right*). Total cognitive load was calculated by summing the scores across these dimensions; higher scores indicated a greater total cognitive load. In this experiment, the McDonald’s *ω* was 0.75.

#### 2.3.4 Working memory

The symmetry span task was employed to assess working memory capacity (Unsworth et al., 2005). Although relational reasoning is often examined using verbal or conceptual tasks, the symmetry span task was selected because it provides a well-established index of domain-general working memory capacity that is sensitive to simultaneous storage and processing demands. In this task, a 4 × 4 grid of squares was displayed on the screen. Each time a square turned red participants were required to remember its location as accurately as possible. Between each instance of a red square, black-and-white images were presented, and participants were asked to determine whether these images exhibited symmetry along the central axis, achieving a minimum accuracy threshold of 85%. At the conclusion of each trial, a blank 4 × 4 grid appeared. Participants were asked to recall and click on the red squares in the order they were presented. The task consisted of three practice trials followed by 12 formal trials (three trials for each condition involving 2–5 squares). The score for this task was calculated based on the total number of correctly recalled squares across all trials, with a maximum achievable score of 42 points. The symmetry span task also demonstrated acceptable reliability (Cronbach’s *α* = 0.64).

#### 2.3.5 Cognitive style

##### 2.3.5.1 Cognitive reflection

Cognitive reflection measures an individual’s ability to inhibit automatic responses and engage in analytical thinking. We used the seven-item version of the Cognitive Reflection Test developed by Toplak et al. (2014). To correctly answer questions (e.g., “*A racket and a ball cost $1.10 total. The racket costs $1 more than the ball. What is the price of the ball?*”), participants needed to suppress any incorrect answers that immediately appeared in their minds and calculate the correct solution through exerting cognitive efforts. The McDonald’s ω for this experiment was 0.82.

##### 2.3.5.2 Need for cognition

The Need for Cognition Scale assessed an individual’s preference for engaging in or avoiding analytical thinking (Cacioppo and Petty, 1982) using an 18-item scale developed by Cacioppo et al. (1984) (e.g., “*I prefer complex questions to simple ones*”), with each item rated on a 1–7-point scale. The McDonald’s ω for this experiment was 0.90.

##### 2.3.5.3 Need for cognitive closure

The need for cognitive closure refers to a person’s tendency to strive for a definitive answer to a question (regardless of whether the answer is correct) to avoid uncertainty. We used the 15-item scale (e.g., “*I dislike uncertain situations*”) developed by Roets and Van Hiel (2011), with items evaluated on a 1–6-point scale (1 = *strongly disagree*, 6 = *strongly agree*). The McDonald’s ω for this experiment was 0.84.

#### 2.3.6 Fluid intelligence

Fluid intelligence was measured using the short version (12-item) of Raven’s Advanced Progressive Matrices (Arthur et al., 1999). This test presents a series of 3 × 3 matrices, with each cell containing a shape and only the bottom right cell being blank. Participants then needed to observe and infer the pattern of changes in the rows and/or columns of each matrix and select the correct option from among eight options. The task was limited to 11 min, and the maximum score was 12 points.

### 2.4 Procedure

First, both groups of participants completed priming tasks. The distant analogy generation group performed a distant analogy–generation task, whereas the distant analogy evaluation group completed a distant analogy–evaluation task. To control for differences in cognitive resources invested in the priming task, we included one item, “*I exert myself for the task*,” to measure the mental effort for the priming task (Seufert, 2018) on a 7-point Likert scale (1 = *completely wrong*, 7 = *absolutely right*). Following the priming tasks, participants’ mental effort was measured. Subsequently, participants completed the multidimensional relational reasoning task; then, their intrinsic cognitive load, extraneous cognitive load, and germane cognitive load were measured. Finally, the participants completed the Working Memory Capacity Test, the Need for Cognition Scale, the Fluid Intelligence Test, the Need for Cognitive Closure Scale, and the Cognitive Reflection Test in random order. The total duration of the experiment was approximately 90 min. To control the effects of fatigue, the study implemented the following measures: (a) a short break (approximately 2 min) was scheduled between the MRRT and the subsequent tasks and, (b) during the experiment, participants were informed that they could take short breaks as needed between tasks.

It is important to distinguish between the two load-related measures used in the present study. The single-item mental effort rating administered immediately after the priming task was used only to index effort invested during the priming phase and to control for between-condition differences in priming task engagement. By contrast, the multidimensional cognitive load scale administered after the MRRT served as the principal mediator and was intended to capture participants’ perceived load during the relational integration task itself.

### 2.5 Statistical analysis

First, the mediating effect of cognitive load was investigated using Model 4 in PROCESS 4.2 (Hayes, 2017); then, the moderated mediating effect was evaluated using Model 5, which tests whether the moderators influence the direct path from the priming task to relational integration. These analyses were conducted with 5,000 bootstrap resamples and 95% confidence intervals. Effects were considered significant when the corresponding confidence interval did not include zero. The independent variable was priming task type; the dependent variable was relational integration; the mediating variable was the cognitive load score; the moderating variables were fluid intelligence, working memory capacity, and cognitive style (cognitive reflection, the need for cognition, and the need for cognitive closure); and the covariate was the priming task’s mental effort rating. Because five moderation tests were conducted, we additionally controlled for multiple testing using the Benjamini–Hochberg false-discovery rate (FDR) procedure. Both the original *p*-values and the FDR-adjusted results were considered when interpreting the moderation effects.

## 3 Results

### 3.1 The relationship between variables

There were no missing data in the final dataset; all 120 participants completed every measure.

The correlation analysis indicated (Table 1) that scores on the relational integration task were significantly negatively correlated with cognitive load scores and significantly positively correlated with fluid intelligence scores, working memory capacity, and cognitive reflection scores. In contrast, no significant correlation with the need for cognition scores or the need for cognitive closure scores was found.

**Table 1:** Correlation matrix.

Variables names	*M* ± *SD*	Reliabilities	1	2	3	4	5	6	7
1 Cognitive load	32.24 ± 6.29	0.75	–						
2 MRRT score	56.01 ± 3.59	0.84	−0.35^***^	–					
3 Fluid intelligence	9.83 ± 1.77	–	−0.20^*^	0.30^***^	–				
4 Working memory capacity	31.66 ± 5.91	0.64	−0.14	0.36^***^	0.49^***^	–			
5 Cognitive reflection	6.51 ± 0.94	0.82	−0.25^**^	0.31^***^	0.35^**^	0.33^***^	–		
6 Need for cognition	77.67 ± 15.61	0.90	−0.14	0.03	−0.07	−0.05	−0.04	–	
7 Need for cognitive closure	61.78 ± 10.28	0.84	0.25^**^	−0.06	−0.21^*^	−0.11	0.01	−0.29^**^	–

*p < 0.05, **p < 0.01, ***p < 0.001.

M, mean; SD, standard deviation.

We then conducted separate correlation analyses for the two priming task groups (Table 2), identical to those performed by Vendetti et al. (2014). In the evaluation group, MRRT scores were significantly correlated with fluid intelligence [*r*_(59)_ = 0.38, *p* = 0.003], working memory capacity [*r*_(59)_ = 0.47, *p* < 0.001], and cognitive reflection scores [*r*_(59)_ = 0.39, *p* = 0.002]. However, for the generation group, the MRRT score showed no significant relationship with fluid intelligence, working memory capacity, or cognitive reflection score. This pattern suggests that the association between individual differences and MRRT performance was less pronounced in the generation group than in the evaluation group.

**Table 2:** Correlation matrix for both conditions.

Variables names	*M_DG_* ± *SD_DG_*	*M_DE_* ± *SD_DE_*	1	2	3	4	5	6	7
1 Cognitive load	30.25 ± 5.96	34.31 ± 5.98	–	−0.21	−0.26	−0.13	−0.32^*^	0.08	0.37^**^
2 MRRT score	56.99 ± 2.18	54.99 ± 4.42	−0.47^***^	–	0.38^**^	0.47^***^	0.39^**^	−0.13	0.03
3 Fluid intelligence	9.85 ± 1.87	9.80 ± 1.68	−0.17	0.25	–	0.52^***^	0.34^**^	−0.09	−0.14
4 Working memory capacity	32.21 ± 5.98	31.09 ± 5.84	−0.10	0.17	0.48^***^	–	0.39^**^	−0.22	−0.01
5 Cognitive reflection	6.64 ± 0.80	6.37 ± 1.05	−0.09	0.02	0.38^**^	0.23	–	−0.10	−0.16
6 Need for cognition	81.25 ± 13.86	73.97 ± 16.56	−0.25	0.16	−0.05	0.09	−0.05	–	−0.37^**^
7 Need for cognitive closure	60.97 ± 9.07	62.61 ± 11.42	0.07	−0.21	−0.28^*^	−0.22	0.30^*^	−0.15	–

DE, distant evaluation; DG, distant generation; M, mean; SD, standard deviation.

The correlations for the distant generation group lie below the diagonal, whereas those for the distant evaluation group lie above the diagonal. *p < 0.05, **p < 0.01, ***p < 0.001.

### 3.2 Independent-samples *t*-test

The independent-samples *t*-test showed no significant difference in scores between the distant analogy–evaluation task (*M* = 34.95, *SD* = 2.22) and the distant analogy–evaluation task (*M* = 35.76, *SD* = 2.06) [*t*_(118)_ = −1.62, *p* = 0.108]. Independent-samples *t* testing also showed that the total score on the MRRT was significantly higher in the generation group (*M* = 56.99, *SD* = 2.18) than in the evaluation group (*M* = 54.99, *SD* = 4.42) [*t*_(118)_ = −3.15, *p* = 0.002, Cohen’s *d* = −0.58], indicating that generating distant analogies could effectively facilitate relational integration compared with merely evaluating distant analogies. The main effects of priming task type on extraneous cognitive load [*t*_(118)_ = 3.53, *p* < 0.001, Cohen’s *d* = 0.65], germane cognitive load [*t*_(118)_ = 1.94, *p* = 0.050, Cohen’s *d* = 0.35], and total cognitive load [*t*_(118)_ = 3.72, *p* < 0.001, Cohen’s *d* = 0.68] were significant, with the cognitive load in the generation condition being less than the cognitive load in the evaluation condition.

Moreover, independent-samples *t* testing showed that the difference in mental effort was significant. The mental effort of the distant generation group (*M* = 3.28, *SD* = 1.51) was significantly lower than that of the distant evaluation group (*M* = 3.95, *SD* = 1.53) [*t*_(118)_ = 2.42, *p* = 0.017, Cohen’s *d* = 0.44]. Participants in the distant evaluation group invested more mental effort; as such, in the subsequent mediation effect and moderated mediation effect analysis, we took the mental effort of the priming task as a covariate.

### 3.3 Mediation analysis

**Figure 1:**
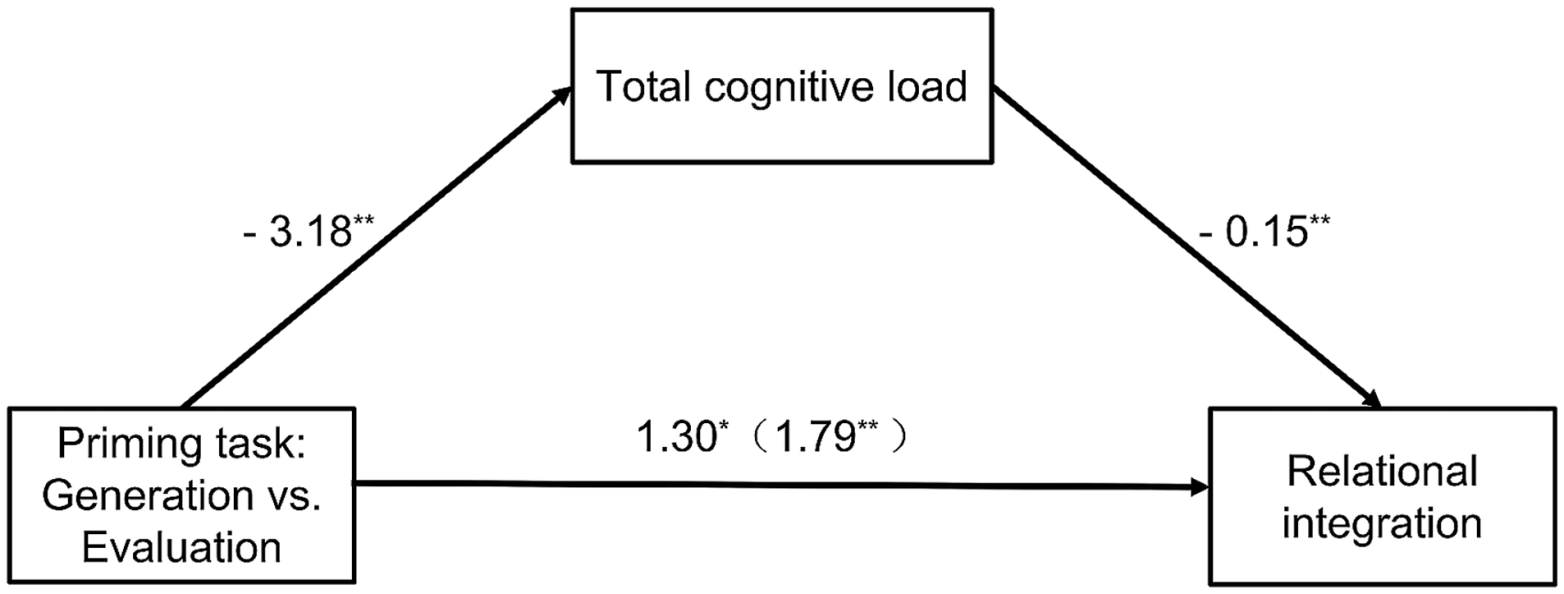
The mediating effect of cognitive load on the relationship between priming tasks and relational integration. *p < 0.05, **p < 0.01.

The results (Table 3) showed that the total effect of the priming task type on relational integration was 1.79 (standard error *(SE)* = 0.65, *t* = 2.78, *p* = 0.006, 95% confidence interval [0.51, 3.07]). Separately, the direct effect was 1.30 (*SE* = 0.65, *t* = 2.00, *p* = 0.048, 95% confidence interval [0.01, 2.59]). Meanwhile, the indirect effect was 0.49, accounting for 27.37% of the total effect (*SE* = 0.20, 95% confidence interval [0.13, 0.93]), indicating that cognitive load had a significant mediating effect and played a partial mediating role. The results of the mediation effect analysis are shown in Figure 1.

**Table 3:** Regression estimates from the mediation model.

Outcome	Predictors	*B*	SE	*t*	*β*
Relational integration	Mental effort	−0.30	0.21	−1.44	−0.13
	Priming task	1.79	0.65	2.78^**^	0.50
	*R*	0.31			
	*R^2^*	0.09			
	*F*	*F*_(2, 117)_ = 6.05, *p* = 0.003			
Cognitive load	Mental effort	1.31	0.34	3.80^***^	0.32
	Priming task	−3.18	1.06	−3.01^**^	−0.51
	*R*	0.45			
	*R^2^*	0.20			
	*F*	*F*_(2, 117)_ = 14.93, *p* < 0.001			
Relational integration	Mental effort	−0.10	0.22	−0.47	−0.04
	Priming task	1.30	0.65	2.00^*^	0.36
	Cognitive load	−0.15	0.06	−2.82^**^	−0.27
	*R*	0.39			
	*R^2^*	0.15			
	*F*	*F*_(3, 116)_ = 6.92, *p* < 0.001			

SE, standard error. *p < 0.05, **p < 0.01, ***p < 0.001.

### 3.4 Moderated mediation analysis

#### 3.4.1 Moderator variable 1: fluid intelligence

The results indicated (Table 4) that fluid intelligence has a marginally significant moderating effect on the association between the priming task type and relational integration (*p* = 0.052). The FDR-adjusted *p*-value was 0.087 for fluid intelligence. Simple slope analysis suggested a trend that the generation task enhanced relational integration for individuals with 1*SD* values below the mean of fluid intelligence (*B* = 2.54, *t* = 2.94, *p* = 0.004), but not for those with 1*SD* values above the mean (*B* = 0.22, *t* = 0.26, *p* = 0.792). Given the non-significant FDR-adjusted *p*-value, this pattern should be interpreted with caution as a suggestive trend rather than a definitive moderation effect.

**Table 4:** Regression estimates from the moderated mediation model (fluid intelligence).

Outcome	Predictors	*B*	SE	*t*
Cognitive load	Mental effort	1.31	0.34	3.80^***^
	Priming task	−3.18	1.06	−3.01^**^
	*R*	0.45		
	*R^2^*	0.20		
	*F*	*F*_(2, 117)_ = 14.93, *p* < 0.001		
Relational integration	Mental effort	−0.30	0.21	−1.42
	Priming task	1.38	0.62	2.23^*^
	Cognitive load	−0.09	0.06	−1.68
	Fluid intelligence	1.61	0.55	2.91^**^
	Priming task × fluid intelligence	−0.65	0.33	−1.96
	*R*	0.50		
	*R^2^*	0.25		
	*F*	*F*_(5, 114)_ = 7.53, *p* < 0.001		

Conditional direct effect	*B*	SE	LLCI	ULCI
*M* − 1*SD*	2.54	0.86	0.83	4.25
*M*	1.38	0.62	0.16	2.61
*M* + 1*SD*	0.22	0.86	−1.45	1.90

LLCI, lower limit confidence interval; M, mean; SD, standard deviation; SE, standard error; ULCI, upper limit confidence interval. *p < 0.05, **p < 0.01, ***p < 0.001.

#### 3.4.2 Moderator variable 2: working memory

The results showed (Table 5) that working memory did significantly moderate the relationship between the priming task type and relational integration (*p* = 0.005). The FDR-adjusted *p*-value was 0.023 for working memory.

**Table 5:** Regression estimates from the moderated mediation model (working memory).

Outcome	Predictors	*B*	SE	*t*
Cognitive load	Mental effort	1.31	0.34	3.80^***^
	Priming task	−3.18	1.06	−3.01^**^
	*R*	0.45		
	*R^2^*	0.20		
	*F*	*F*_(2, 117)_ = 14.93 *p* < 0.001		
Relational integration	Mental effort	−0.20	0.20	−0.97
	Priming task	1.14	0.60	1.91
	Cognitive load	−0.12	0.05	−2.40^*^
	WM	0.62	0.15	4.03^***^
	Priming task × WM	−0.28	0.10	−2.90^**^
	*R*	0.55		
	*R^2^*	0.30		
	*F*	*F*_(5, 114)_ = 9.89, *p* < 0.001		

Conditional direct effect	*B*	SE	LLCI	ULCI
*M* − 1*SD*	2.79	0.83	1.15	4.43
*M*	1.14	0.60	−0.04	2.32
*M* + 1*SD*	−0.51	0.82	−2.13	1.12

LLCI, lower limit confidence interval; M, mean; SD, standard deviation; SE, standard error; ULCI, upper limit confidence interval; WM, working memory. *p < 0.05, **p < 0.01, ***p < 0.001.

**Figure 2:**
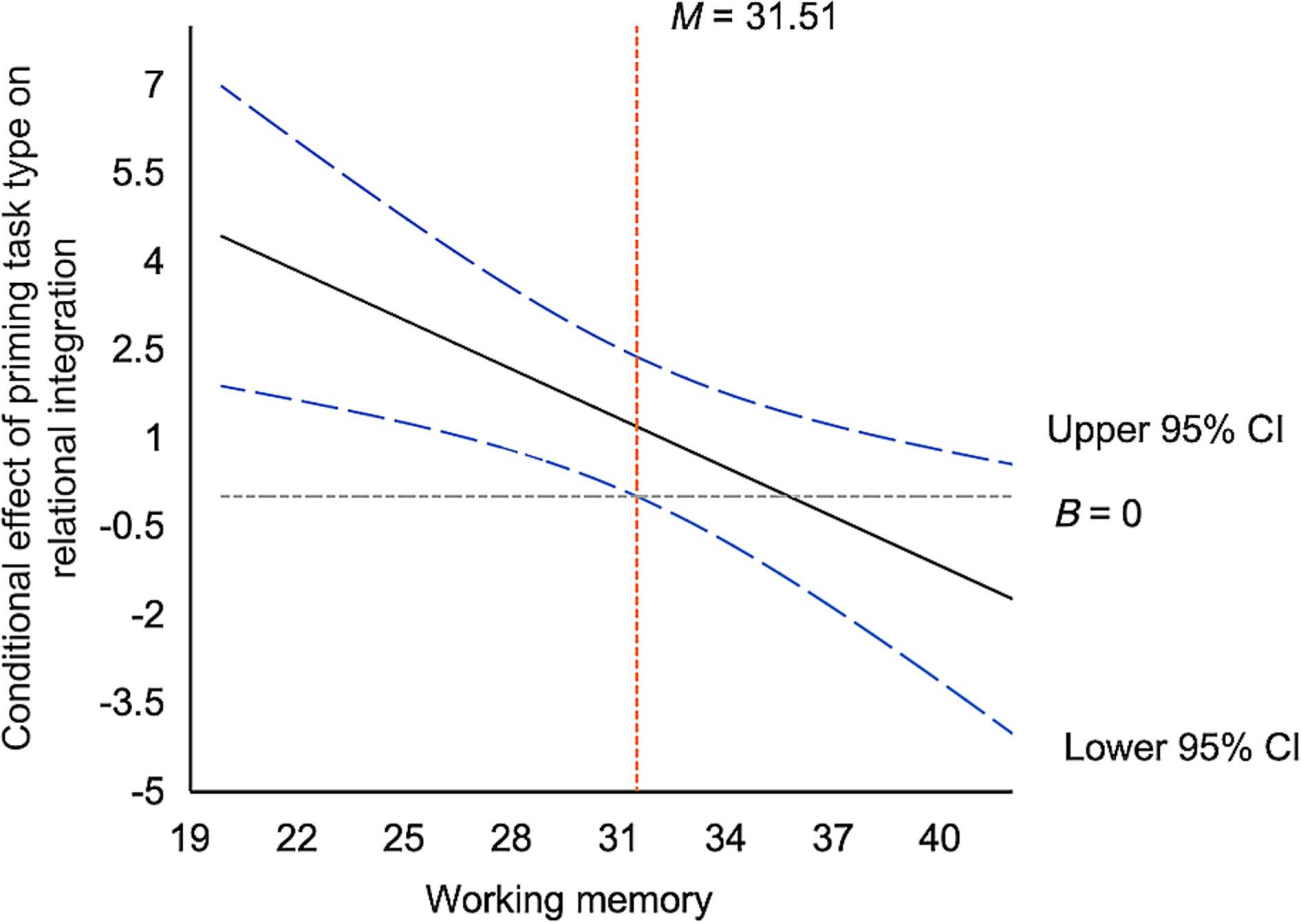
Johnson–Neyman plot of the moderating effect of working memory. In the graph, the black line represents the moderated effect, and the blue band shows the 95% confidence interval around that effect. The horizontal gray dashed line indicates the reference point where the effect is zero. The vertical orange dashed lines mark the threshold values.

To further analyze the moderating effect of working memory on the relationship between priming task type and relational integration, we conducted a simple slope analysis. The analogy-generation task significantly facilitated relational integration for individuals with 1*SD* values below the mean of working memory capacity (*B* = 2.79, *t* = 3.36, *p* = 0.001). In contrast, for individuals with 1*SD* values above the mean of working memory capacity, the facilitation effect was not significant (*B* = −0.51, *t* = −0.62, *p* = 0.539). Johnson–Neyman analysis indicated that the effect of priming task type on relational integration was significant only at a relatively lower working memory capacity (score ≤ 31.51 points), covering approximately 49.17% of the sample (see Figure 2). However, when the working memory capacity exceeded 31.51 points, completing either the distant analogy–generation task or the distant analogy–evaluation task beforehand had no significant difference in its impact on subsequent relational integration performance.

#### 3.4.3 Moderator variable 3: cognitive reflection

The results also revealed (Table 6) that cognitive reflection significantly moderated the relationship between the priming task type and relational integration (*p* = 0.037). The FDR-adjusted *p*-value was 0.087 for cognitive reflection. Simple slope analysis indicated that, for individuals with 1*SD* values below the mean of cognitive reflection, there was a positive effect on relational integration (*B* = 2.60, *t* = 2.82, *p* = 0.006), whereas the effect was not significant for those with 1*SD* values above the mean (*B* = 0.58, *t* = 0.85, *p* = 0.398). Given the FDR-adjusted threshold, we interpret this as a suggestive trend consistent with our hypothesis, but one that requires replication in future studies with larger samples.

**Table 6:** Regression estimates from the moderated mediation model (cognitive reflection).

Outcome	Predictors	*B*	SE	*t*
Cognitive load	Mental effort	1.31	0.34	3.80^***^
	Priming task	−3.18	1.06	−3.01^**^
	*R*	0.45		
	*R^2^*	0.20		
	*F*	*F*_(2, 117)_ = 14.93, *p* < 0.001		
Relational integration	Mental effort	−0.07	0.21	−0.31
	Priming task	1.28	0.63	2.03^*^
	Cognitive load	−0.12	0.05	−2.19^*^
	CR	2.83	0.98	2.89^**^
	Priming task × CR	−1.41	0.67	−2.11^*^
	*R*	0.48		
	*R^2^*	0.23		
	*F*	*F*_(5, 114)_ = 6.85, *p* < 0.001		

Conditional direct effect	*B*	SE	LLCI	ULCI
*M* − 1*SD*	2.60	0.92	0.77	4.43
*M*	1.28	0.63	0.03	2.52
*M* + 1*SD*	0.58	0.69	−0.78	1.94

CR, cognitive reflection; LLCI, lower limit confidence interval; M, mean; SD, standard deviation; SE, standard error; ULCI, upper limit confidence interval. *p < 0.05, **p < 0.01, ***p < 0.001.

#### 3.4.4 Moderator variable 4: need for cognition

The need for cognition had no significant moderating effect on the direct path of the mediation model (Table 7).

**Table 7:** Regression estimates from the moderated mediation model (need for cognition).

Outcome	Predictors	*B*	SE	*t*
Cognitive load	Mental effort	1.31	0.34	3.80^***^
	Priming task	−3.18	1.06	−3.01^**^
	*R*	0.45		
	*R^2^*	0.20		
	*F*	*F*_(2, 117)_ = 14.93, *p* < 0.001		
Relational integration	Mental effort	−0.10	0.22	−0.46
	Priming task	1.40	0.67	2.10^*^
	Cognitive load	−0.15	0.06	−2.66^**^
	NFC	−0.07	0.06	−1.09
	Priming task × NFC	0.04	0.04	0.92
	*R*	0.40		
	*R^2^*	0.16		
	*F*	*F*_(5, 114)_ = 4.39, *p* = 0.001		

NFC, need for cognition; SE, standard error. *p < 0.05, **p < 0.01, ***p < 0.001.

#### 3.4.5 Moderator variable 5: need for cognitive closure

The need for cognitive closure had no significant moderating effect on the direct path of the mediation model (Table 8). These results remained non-significant after FDR correction, indicating that these two dispositional cognitive styles do not influence the extent to which analogy generation facilitates relational integration.

**Table 8:** Regression estimates from the moderated mediation model (need for cognitive closure).

Outcome	Predictors	*B*	SE	*t*
Cognitive load	Mental effort	1.31	0.34	3.80^***^
	Priming task	−3.18	1.06	−3.01^**^
	*R*	0.45		
	*R^2^*	0.20		
	*F*	*F*_(2, 117)_ = 14.93, *p* < 0.001		
Relational integration	Mental effort	−0.10	0.22	−0.48
	Priming task	1.25	0.65	1.92
	Cognitive load	−0.17	0.06	−2.99^**^
	NFCC	0.14	0.09	1.46
	Priming task × NFCC	−0.09	0.06	−1.41
	*R*	0.41		
	*R^2^*	0.17		
	*F*	*F*_(5, 114)_ = 4.58, *p* = 0.001		

NFCC, need for cognitive closure; SE, standard error.

## 4 Discussion

The present study examined whether generating, rather than evaluating, distant analogies is associated with better subsequent relational integration and whether this effect depends on individual differences in cognitive abilities and cognitive styles. Overall, the results suggest that generation was associated with better MRRT performance than evaluation, that this difference was partially mediated by cognitive load, and that the direct effect varied across individuals.

### 4.1 The relationship between variables

The MRRT score was strongly linked to fluid intelligence, working memory, and cognitive reflection but not to the other two self-assessed cognitive styles. This suggests that fluid intelligence, working memory, and cognitive reflection may primarily influence relational reasoning (Campitelli and Gerrans, 2014; Gray and Holyoak, 2020).

The MRRT score was negatively correlated with the cognitive load score, suggesting that a lower cognitive load score may be associated with higher relational reasoning performance.

Fluid intelligence and working memory showed a substantial connection, implying a close association (Foster et al., 2015). In line with earlier research (Brañas-Garza et al., 2012; Hanaki et al., 2016), we discovered a moderate association between fluid intelligence and cognitive reflection. Individuals who could resist the need to adopt “obvious” solutions would be better able to develop and maintain goals, as well as generate proper conclusions. The need for cognitive closure was found to be positively connected with cognitive load. When individuals are less anxious to find a definitive answer, they perceive a lower cognitive load.

Previous research has demonstrated that the distant analogy–generation task can temporarily break the link between fluid intelligence and relational integration (Andrews and Bohadana, 2018; Vendetti et al., 2014). One possible explanation for this phenomenon is that the facilitation effect varies among individuals. Those with lower cognitive abilities are more likely to benefit from priming tasks, thereby enhancing their performance to a level closer to that of individuals with higher cognitive abilities.

### 4.2 The effect of priming task type on relational integration and cognitive load

The independent-samples *t*-test revealed that participants in the generation condition scored substantially higher on the MRRT than those in the evaluation condition and reported markedly lower total cognitive, extraneous cognitive, and marginally lower germane cognitive loads. These findings support H1a (improved relational integration) and H1b (reduced cognitive load). Notably, the effect sizes were moderate to large for total cognitive load (Cohen’s *d* = 0.68) and extraneous cognitive load (Cohen’s *d* = 0.65), suggesting that the reduction in extraneous load was a key driver of the overall cognitive load reduction.

Our results also revealed that the generation task required considerably less mental effort than the evaluation task. This pattern is consistent with the idea that active generation may engage prior relational knowledge and orient participants toward a more relational mode of processing, thereby reducing the effort required in the subsequent task. Notably, the present interpretation does not assume that generation is intrinsically easier than evaluation at the priming stage. Rather, it suggests that the more effortful constructive processing involved in generation may have functioned as preparatory relational work, thereby reducing the amount of relational search and integration required in the subsequent MRRT.

The observed reduction in total cognitive load is broadly consistent with Cognitive Load Theory in the sense that participants in the generation condition may have entered the subsequent reasoning task in a more relationally prepared state. However, because cognitive load was assessed through post-task self-reporting rather than online process measures, the present data should not be considered strong evidence regarding the precise distribution of intrinsic, extraneous, and germane load.

### 4.3 The mediating role of cognitive load

Having established that generating distant analogies leads to improved relational integration and reduced cognitive load, we tested whether cognitive load serves as a mediator of this facilitation effect (H2). The mediation analysis revealed a marked indirect effect of priming task type on MRRT scores via cognitive load, accounting for 27.37% of the total effect. This finding supports H2 and successfully replicates and extends previous work by Du and Sun (2022). The mediating effect of cognitive load suggests that the difference between generation and evaluation was not limited to a direct performance advantage. Instead, participants who generated distant analogies subsequently reported lower cognitive loads during the MRRT, which in turn was associated with better relational integration performance. This is consistent with the interpretation by Vendetti et al. (2014). The generation condition prompted participants to engage in more relational processing of the inputs. In other words, participants might stop scanning for surface features (such as colors, forms, and specific features) and start looking for abstract schemas stripped of such specifics, thereby freeing working memory resources through more efficient representations of the inputs. This kind of explanation of lower cognitive load might be due to participants’ entering a mode of processing the inputs and generating abstract schemas to represent them, a key signature of analogical encoding (Gentner et al., 2003; Goldwater and Jamrozik, 2019), and the generation of visuospatial abstract schemas that play a role in analogical transfer (Kubricht et al., 2017; Navarrete-Ulloa and Trench, 2025).

Previous research has indicated that imposing cognitive load on college students diminishes their capacity for making relational choices (Waltz et al., 2000). Consequently, alleviating participants’ cognitive load enhanced their ability to engage in relational decision-making, thereby supporting the principles of Cognitive Load Theory. The present findings suggest that generating, rather than merely evaluating, distant analogies reduces learners’ cognitive load, thereby freeing up working memory capacity and enhancing their ability to integrate relational information in a subsequent task.

These findings also align with the broader notion that self-generated processing is more effective for learning and retention than passive processing. Slamecka and Graf (1978) termed this the “generation effect”—a robust phenomenon whereby actively producing a response leads to better memory and deeper cognitive processing compared with simply reading or evaluating a prespecified response. In the present study, the generation task required participants to actively construct a missing D term (e.g., “stomach: _”) and, in doing so, to identify an abstract higher-order relation that unifies two semantically distant domains. This generative process likely engages participants in deeper semantic elaboration and schema construction, which in turn reduces the cognitive resources required to process relational information later on. In contrast, the evaluation task presented a complete analogical relationship (e.g., “stomach: food”), allowing participants to verify the relation without engaging in the same level of relational construction. Consequently, the evaluation group experienced higher cognitive load during the priming phase, which then carried over to reduce their subsequent relational integration performance.

Overall, the marked mediation effect supports the interpretation that cognitive load reduction—driven by the active and generative nature of constructing distant analogies—is a key mechanism through which the priming task facilitates relational integration.

### 4.4 The moderating effects of fluid intelligence, working memory, and cognitive reflection on the mediation model

Fluid intelligence, working memory, and cognitive reflection were all significant predictors of relational integration performance. Fluid intelligence involves the formulation and maintenance of goals within working memory; the inference of relationships; and, ultimately, the execution of judgments. Consistent with previous research, our findings indicated that fluid intelligence strongly predicts relational processing (Kubricht et al., 2017; Vendetti et al., 2014). Relational thinking process requires us to simultaneously encode multiple associations in working memory, integrate these connections into a coherent structure, and adjust the matching of relational representations (Richland et al., 2006). Our results demonstrated that working memory positively predicts relational integration, aligning with earlier studies. In agreement with Gray and Holyoak (2020), we found that cognitive reflection influences relational integration. Accurate relational judgments necessitate integrating relationships among items while minimizing interference from other potentially misleading interactions. Consequently, a high level of cognitive reflection fosters relational integration.

Regarding the moderating effect of individual differences, only the moderating effect of working memory remained significant after controlling for multiple comparisons. Thus, only H4 was supported after correction, whereas H3 and H5 were not supported. Working memory substantially moderated the direct effect of the priming task type on relational integration. Notably, cognitive load and working memory are theoretically closely linked. According to Cognitive Load Theory (Sweller, 2010; Sweller et al., 2019), working memory capacity is the primary bottleneck for processing complex relational information: when the total cognitive load exceeds available working memory resources, performance deteriorates. Generating distant analogies reduces total cognitive load (as shown in Sections 3.2 and 4.2), thereby freeing up working memory resources that can be redirected to relational integration. This mechanism is particularly beneficial for individuals with lower working memory capacity, as they are more susceptible to cognitive overload. Halford et al. (1998, 2010) likewise argued that relational integration inherently requires the simultaneous binding of multiple relations in working memory and that individual differences in working memory capacity directly constrain the ability to handle such complexity. Therefore, the reduction of cognitive load achieved by distant analogy generation may compensate for limited working memory resources, explaining why the facilitation effect was notable only for participants with lower working memory capacity (see Section 3.4.2).

More cautiously, the present findings suggest that the beneficial effect of the analogy-generation task may be especially pronounced for individuals with more limited cognitive resources, although this pattern was robust only for working memory capacity after correction. In this respect, our findings are broadly consistent with prior studies suggesting that relational support or analogy-based interventions may be particularly helpful for lower-ability individuals. A possible explanation is that individuals with higher cognitive ability may already be more likely to focus on relational structure (Vendetti et al., 2014) and to possess a broader repertoire of effective cognitive strategies (Gonthier and Thomassin, 2015). As a result, they may already perform relatively well even in the evaluation condition, leaving less room for improvement through the generation manipulation. Nevertheless, given that only the working memory effect remained significant after FDR correction, the present study provides the strongest evidence for the moderating role of working memory capacity rather than for a more general lower-ability advantage.

In contrast, neither of the other two cognitive styles—need for cognition and need for cognitive closure—predicted relation integration; moreover, their interactions with priming task type did not predict relation integration scores, meaning they did not markedly moderate the direct path of the mediation model. As a result, H6 and H7 were not supported, contrary to our expectations. The need for cognition might be expected to positively influence relational integration by promoting increased effort. However, consistent with the works of Grossnickle et al. (2016) and Gray and Holyoak (2020), our study found no evidence supporting the notion that the need for cognition predicts performance in relational reasoning tasks. This pattern may indicate that stable preferences for effortful thinking or certainty-seeking are less relevant to the short-term impact of a brief relational priming manipulation than are core cognitive resources such as working memory capacity. One possible explanation was that the MRRT presented challenges exceeding individuals’ general inclination toward engagement in and enjoyment of analytical thinking.

We found that cognitive load accounted for approximately 30% of the variance observed in the facilitation effect. The results indicated that, among the internal mechanisms contributing to this facilitation effect, the mediating pathway represented one-third of the total effect value across all participants; conversely, for individuals with lower cognitive abilities, the direct pathway constituted two-thirds of their total effect value. Taken together, these findings suggest that distant analogy–generation tasks predominantly benefit those with lower cognitive abilities. The present findings also clarify how this study extends our earlier work (Du and Sun, 2022). Whereas the earlier study primarily established that distant analogy generation can improve relational integration performance, the current study provides a more fine-grained explanation by validating the mediating mechanism and testing multiple individual-difference moderators. In this sense, the present study moves beyond merely demonstrating the effect itself and offers a more detailed account of its cognitive basis and its boundary conditions.

## 5 Limitations and future research

Although this study explored the factors influencing the facilitation effect through a review of prior literature and yielded some new findings, several limitations should be acknowledged, and future research directions are suggested.

First, some limitations concern the measurement and interpretation of individual differences. Self-reported measurements of cognitive style may not accurately reflect behavioral tendencies, and caution is warranted when interpreting the results. Individuals with poorer analytical thinking abilities (as assessed by cognitive reflection) sometimes exhibit miscalibration of cognitive demands, particularly when their behavioral performance contradicts their self-reported perceptions—they may indeed overestimate their cognitive demands (Pennycook et al., 2017). Behavioral measurement methods for cognitive styles, such as basic ratio or heuristic problem-solving tasks, can be employed to clarify the actual contribution of cognitive style to relational reasoning (Stanovich and West, 2008).

Relatedly, we did not discover a relationship between self-reported cognitive style scores (need for cognition and need for cognitive closure) and relational integration in our study. However, previous research has reported that such a relationship exists in other types of reasoning tasks, including probabilistic reasoning (Viator et al., 2020), syllogistic and mathematical reasoning (Macpherson and Stanovich, 2007; Setiawan, 2020), and heuristic tasks (Toplak et al., 2011). This discrepancy suggests that relational reasoning may not be a unitary construct and that the relevance of cognitive style may depend on the specific type of reasoning task involved. Future studies should therefore examine more carefully how different dimensions of relational reasoning relate to cognitive abilities and cognitive styles.

Second, several methodological limitations should be noted. Most importantly, the study compared distant analogy generation only with distant analogy evaluation and did not include a no-priming control condition. As a result, the present findings cannot suggest whether generation improved relational integration relative to baseline, whether evaluation suppressed performance, or whether both processes contributed to the observed difference. In addition, participants completed several cognitive tasks within a 90-min session; as such, fatigue may have influenced later performance. Although efforts were made to reduce fatigue, this possibility cannot be ruled out. Future studies could strengthen the design by including a no-priming baseline condition, shortening the testing session, or more extensively counterbalancing task order.

A further methodological issue concerns the relatively high MRRT scores observed in the present sample, suggesting a mild ceiling tendency. Although this tendency did not appear to undermine the main findings, some compression at the upper end of the score range may have attenuated certain correlations or interaction effects, particularly among higher-performing participants. One possible reason is that the task may have been relatively easy for this sample, which consisted of students from a highly selective university in China. Future research could address this issue by recruiting participants with a broader range of cognitive ability and by incorporating more difficult items to better capture individual differences across the full performance spectrum.

Finally, the present findings also point to several directions for future research and application. From an instructional perspective, the findings tentatively suggest that brief pre-task activities requiring students to actively generate distant analogies may help prepare some learners—especially those with lower working memory capacity—for subsequent tasks involving relational integration, who may otherwise be less likely to spontaneously prioritize relational structure. However, because the present study did not include a no-priming control condition and was conducted outside authentic instructional contexts, these implications should be considered preliminary. Future research should test whether similar effects can be observed in classroom-based learning environments.

Beyond analogy generation, other forms of generative activities may also promote relational reasoning. For example, asking children to explain why a relational match is correct—rather than simply reporting the match—has been shown to increase their subsequent preference for relational over object similarity (Brockbank et al., 2023). Although that study focused on young children and did not measure cognitive load, its findings align with the broader principle that active generation facilitates relational thinking. Future research could examine whether such explanation-based generation tasks operate through similar cognitive load reduction mechanisms and whether they can be effectively combined with analogy-generation tasks across different age groups and educational levels.

## 6 Conclusion

In summary, our findings suggest that generating, rather than merely evaluating, distant analogies is associated with better subsequent relational integration performance. This difference was partially mediated by lower cognitive load during the later relational integration task and was more evident among some individuals than others, particularly those with relatively lower working memory capacity. Overall, the findings support the view that the benefits of distant analogy generation are not uniform across learners and should be understood in relation to cognitive processing demands and individual differences.

## Data Availability

The raw data supporting the conclusions of this article will be made available by the authors, without undue reservation.

## Footnotes

1
https://jakewestfall.shinyapps.io/pangea/

2
http://lsa.colorado.edu/
